# Exposure to Light at Night and Risk of Cancer: A Systematic Review, Meta-Analysis, and Data Synthesis

**DOI:** 10.3390/cancers16152653

**Published:** 2024-07-26

**Authors:** Samuel Ma, Yossef Alsabawi, Hashem B El-Serag, Aaron P Thrift

**Affiliations:** 1 School of Medicine, Baylor College of Medicine, Houston, TX 77030, USA; samuel.ma@bcm.edu; 2 School of Medicine, University of Texas Rio Grande Valley, Edinburg, TX 78539, USA; yossef.alsabawi01@utrgv.edu; 3 Section of Gastroenterology and Hepatology, Department of Medicine, Baylor College of Medicine, Houston, TX 77030, USA; hasheme@bcm.edu; 4 Veterans Affairs Health Services Research and Development Service Center for Innovations in Quality, Effectiveness, and Safety (IQuESt), Michael E. DeBakey Veterans Affairs Medical Center, Houston, TX 77030, USA; 5 Section of Epidemiology and Population Sciences, Department of Medicine, Baylor College of Medicine, Houston, TX 77030, USA; 6 Dan L Duncan Comprehensive Cancer Center, Baylor College of Medicine, Houston, TX 77030, USA

**Keywords:** light at night, cancer incidence, systematic review, meta-analysis, breast cancer

## Abstract

**Simple Summary:**

Cancer is the second leading cause of death globally, and the causes of cancer are numerous and multifactorial, including genetic and environmental factors. There is a developing interest in the role of sleep and light at night (LAN) exposure in cancer development. The aim of our study is to further understand the relationship between LAN exposure and the risk of cancer by systematically reviewing existing literature assessing LAN exposure and the risk of various cancer types. We found a positive association between LAN exposure and breast cancer risk, but there was insufficient data to convincingly draw a conclusion for other cancer types. This emphasizes the need for further research not only assessing LAN exposure and the incidence of other cancers but also the pathophysiology of sleep interruption on the formation of cancer.

**Abstract:**

Background: Emerging interest surrounds the role of environmental factors, notably exposure to light at night (LAN), as a potential cause of cancer. The aim of this study was to conduct a systematic review and, if possible, meta-analysis of observational studies on LAN and cancer risk of multiple types. Methods: A systematic literature search in PubMed, Web of Science, and Embase, spanning from inception to May 2023, was conducted. Studies focusing on the association between LAN exposure and cancer risk in adult populations were included. We used random effects models to calculate pooled risk estimates (RR) and 95% confidence intervals (CI). We assessed study quality using the Risk of Bias in Non-randomized Studies of Interventions. Results: Among 8492 initially identified studies, 26 met the inclusion criteria (13 were case–control and 13 were cohort studies). These studies were published from 2001 to 2023 and assessed diverse cancer types in North America, Asia, Europe, and Australia. Except for breast cancer, there was a paucity of site-specific cancer studies. In the meta-analysis of 19 breast cancer studies, higher exposure to indoor (summary RR, 1.08; 95% CI 1.01–1.15) and outdoor (summary RR, 1.10; 95% CI, 1.04–1.15) LAN were associated with increased risk. After excluding one low-quality study, the results were unchanged. Conclusions: We found a positive association between LAN exposure and breast cancer risk in women. However, data are lacking for other cancer types, and further studies are required to better understand the role of LAN on cancer.

## 1. Introduction

In 2019, cancer as a whole was the second leading cause of death globally, with 10.0 million deaths [1]. Although the most frequently diagnosed cancer type and the leading cause of cancer death vary widely between countries and regions of the world, the three cancers with the highest burden according to disability-adjusted life years for the period 2010 to 2019 were tracheal/bronchus/lung cancer, colorectal cancer, and stomach cancer [1,2]. Breast cancer was the cancer with the largest burden among women globally and fourth overall. The causes of cancer are multifactorial and include genetic, biological, and environmental factors depending on the location of the cancer. While the most common environmental risk factors of cancer include tobacco smoking, heavy alcohol consumption, obesity, and low fruit and vegetable intake [3,4,5], other modifiable and occupational factors have been implicated as carcinogenic [6,7,8,9,10].

Among the environmental risk factors for cancer, exposure to light at night (LAN) and sleep interruption have been of increasing interest. A dramatic increase in urbanization globally in the last several decades, with 80% of the world’s population now living in areas with exposure to LAN, points to an ever-growing need to understand the potential health implications that LAN and disrupted sleep rhythms may have on human health [11]. The International Agency for Research on Cancer in 2010 deemed shift work and LAN as “probably carcinogenic to humans” because of a possible link to multiple tumor types [12]. The exact mechanisms by which LAN exposure may increase an individual’s risk for cancer are unclear. Potential mechanistic factors proposed to date include circadian disruption, oxidative stress, and hormonal disruptions [13,14]. A link between LAN exposure and increased risk for lung cancers, leukemias, and hepatocarcinomas in mice models has already been established, but few observational studies in humans have been performed [15,16]. LAN is typically measured in human studies using satellite images or self-reported measures of indoor/outdoor light exposure. The majority of prior efforts to consolidate results from observational studies of LAN and cancer risk have focused on breast cancer, including three meta-analyses, with no prior systematic review and meta-analysis focused on other tumor types [17,18,19]. In the most recent meta-analysis of LAN and breast cancer risk by Urbano et al., including 17 studies published through September 2021, the highest level of LAN exposure had a positive association with breast cancer risk compared to the lowest level of LAN exposure (risk Ratio [RR], 1.11; 95% confidence interval [CI] 1.07–1.15) regardless of study design and method of LAN assessment [17].

Furthering our knowledge of the relationships between LAN exposure and cancer risk of multiple types is important in terms of public health policy, including cancer prevention and education. The aim of this study was to undertake a systematic review and, if possible, meta-analysis of observational studies of the association between LAN exposure and cancer risk.

## 2. Methods

This study was reported in accordance with the Preferred Reporting Items for Systematic Reviews and Meta-Analyses (PRISMA) statement [20] and is consistent with the Meta-analysis of Observational Studies in Epidemiology (MOOSE) guidelines for the meta-analysis of observational studies. This study has not been registered [21].

### 2.1. Search Strategy

We conducted a systematic literature search from the inception date to 31 May 2023 of PubMed, Web of Science, and Embase for all studies reporting the association between LAN exposure and the risk of cancer. The detailed search strategies used for each of the three databases are shown in Supplementary Table S1. Searches were restricted to human studies with an English language title and abstract. Additionally, we reviewed references from relevant papers to identify further eligible studies. 

### 2.2. Selection Criteria 

Studies were included if they met the following criteria: (1) study design: case–control, cohort, or cross-sectional studies; (2) study population: adult (≥18 years) population; (3) exposures: exposure to LAN assessed through indoor and/or outdoor exposure to lighting sources; (4) outcomes: cancer risk, with reported risk estimates or data available to compute the associated risk estimates. If multiple articles were published using identical populations, we used the study with the most complete data on the largest number of participants in a specific analysis. Only full-text published original data were used for reporting or analyses. Of note, accepted studies using self-reported LAN exposure defined it using subjective terms such as low, medium, or high or other factors such as the use of a nightlight or an open window with light coming from outside. Studies that assessed shift work as an exposure group without explicitly including LAN as a variable in the analysis were not included in our study. 

Criteria for exclusion were: (1) study design (e.g., qualitative studies, abstracts, and posters); (2) studies conducted in a pediatric population (<18 years); (3) studies with only benign conditions as the outcome; (4) studies with only mortality outcomes.

After the removal of duplicates, each title and abstract were reviewed independently by two authors using the Rayyan software platform (https://rayyan.ai/cite) [22]. Discrepancies were noted and resolved by consensus between the two authors and others, if required, based on the specific inclusion and exclusion criteria outlined above. The full-text articles for the abstracts that passed the initial screen were then examined by two separate authors to assess final eligibility for our analysis based strictly on the criteria stated above.

### 2.3. Data Extraction 

For articles that met the inclusion criteria, two independent reviewers used standardized data collection forms to extract the following from each study: first author, publication year, journal, country or region where the study was performed, study design, sampling frame, participant source, LAN assessment method (e.g., satellite data or self-reported exposure from outside light or indoor lighting), cancer type, reference group, exposure, un/adjusted odds ratios, hazard ratios or relative risks with a 95% CI, adjusted or matched factors, and, if appropriate, reason for exclusion following full-text review. Any disparities between the reviewers regarding data extraction were resolved by referring to the original studies.

### 2.4. Quality Assessment 

The risk of bias was independently assessed by two authors using the Risk of Bias in Non-randomized Studies of Interventions (ROBINS-I) tool (Supplementary Table S2) [23]. ROBINS-I uses three steps to evaluate the level of bias in non-randomized studies: (1) presenting the review question, potential confounders, co-interventions, and exposure and outcome measurement accuracy; (2) describing each study as a hypothetical target experiment and the specific confounders and co-interventions that would be associated with it; (3) assessment of the risk of bias in 7 categories: (1) bias due to confounding, (2) bias due to selection of participants, (3) bias in classification of exposures, (4) bias due to departures from intended exposures, (5) bias due to missing data, (6) bias in measurement of outcomes, and (7) bias in selection of reported results. For each study, each item was judged to be at a low, moderate, serious, or critical risk of bias, which was then followed up by an overall judgment of the study’s risk of bias. Discrepancies were resolved by consensus.

### 2.5. Statistical Analysis

The meta package implemented in Stata version 14 (StataCorp., College Station, TX, USA) was used to calculate pooled RRs (compiling available risk estimates from the individual studies) and associated 95% CIs using random effects models due to clinical heterogeneity between studies. Analysis was carried out on adjusted RRs when available or unadjusted RRs otherwise. Statistical heterogeneity was assessed using the I^2^ statistic [24], with I^2^ > 25% indicating moderate statistical heterogeneity and I^2^ > 50% indicating a substantial level of heterogeneity between studies.

### 2.6. Data Synthesis 

We aimed to undertake a meta-analysis if there were sufficient data. However, if this was not possible, the plan was to conduct a narrative synthesis to explore, describe, and interpret the available evidence for associations of LAN exposure with cancer risk.

## 3. Results

**Figure 1:**
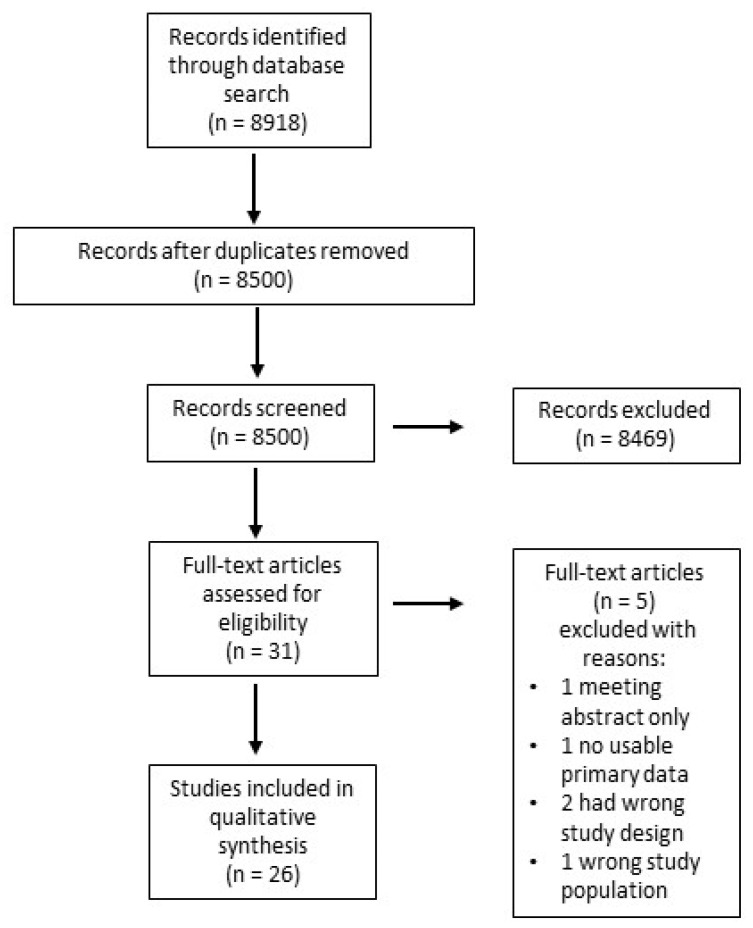
Flow chart depicting the search strategy and subsequent selection criteria for studies included in the analysis.

The search strategy identified 8918 studies. Following the removal of 418 duplicates, 8500 studies underwent title and abstract review. Of these, 31 were identified for full-text evaluation. Of the 31 articles chosen for full review, we excluded 5 for having abstract only (*n* = 1), no usable primary data *(n* = 1), the wrong study design (*n* = 2), and the wrong study population (*n* = 1). Therefore, 26 studies were included in the final review (Figure 1). 

The studies were published from 2001 to 2023. Geographically, the studies assessed populations in North America (*n* = 16) [25,26,27,28,29,30,31,32,33,34,35,36,37,38,39,40], Asia (*n* = 4) [41,42,43,44], Europe (*n* = 4) [45,46,47,48], and Australia (*n* = 2) [49,50]. The vast majority of these papers included breast cancer as an outcome (*n* = 19), while colorectal cancer (*n* = 2), prostate cancer (*n* = 1), thyroid cancer (*n* = 1), pancreatic cancer (*n* = 1), non-Hodgkin’s lymphoma (*n* = 1), liver cancer (*n* = 1), and endometrial cancer (*n* = 1) were represented in a total of eight studies. One study, Garcia-Saenz 2018, included data separately for both risks of breast and prostate cancers. Table 1 describes all studies and their associated study designs. Thirteen were case–control, and thirteen were cohort studies. Supplementary Table S3 contains additional details. Ten studies reported associations with indoor LAN, twelve with outdoor LAN, and four reported associations separately for both indoor and outdoor LAN.

Risk of bias assessment (Supplementary Table S4) found eight studies (29.6%) to be at moderate risk of bias due to confounding because of a failure to control for other cancer risk factors or other environmental disruptors of circadian rhythm [27,42,43,44,46,47,48,49]. Examples of failure to control for other cancer risk factors include the failure to record estrogen or progesterone receptor positivity, family history of cancer, prior radiation, family history of breast cancer, postmenopausal hormone use, or smoking history. The risk of bias due to selection was judged to be moderate for eight studies due to small sample sizes, poorly generalizable populations, or unmatched cases [25,30,33,34,40,41,44,48]. Three studies (11.1%) were judged to be at critical risk of bias due to selection because of non-randomly selected controls or a collection of multiple moderate risk factors [42,43,49]. Regarding exposure measurement, 11 studies (40.7%) were judged to be at a moderate risk of bias. These studies were found to have utilized indirect measures of LAN, non-intuitive categories of light exposure, or solely self-report surveys [25,27,29,34,35,39,40,43,46,48,50]. One study (3.7%) was found to be at critical risk of measurement of exposure bias due to using a non-validated and non-expansive self-administered questionnaire [42]. All other domains were considered at low risk of bias in all studies. Regarding the study-level risk of bias assessment, three studies (11.1%) were determined to be at moderate risk of bias [43,48,49]. Only one study (3.7%) was deemed to be at critical risk of bias overall [42].

### 3.1. Meta-Analysis of LAN and Risk of Breast Cancer

Of the 19 studies on LAN exposure and breast cancer risk, 9 examined only indoor sources of LAN, 6 examined only outdoor sources of LAN, and the other 4 examined both indoor and outdoor sources of LAN (Table 1). Indoor LAN measures were entirely self-reported based on participant answers to survey questions, which included factors such as reading with a light before sleeping, a turned-on TV while sleeping, perceived level of light in the room while sleeping, or sleeping with a night light on. Outdoor LAN in all studies used objective satellite data in units of nanowatt/cm^2^/steradian to measure the level of night-time illumination geographically. These quantitative data were broken down into tertiles, quartiles, or quintiles for exposure groups. One study also incorporated blue light as a measure of exposure to outdoor LAN using satellite data [45]. 

**Figure 2:**
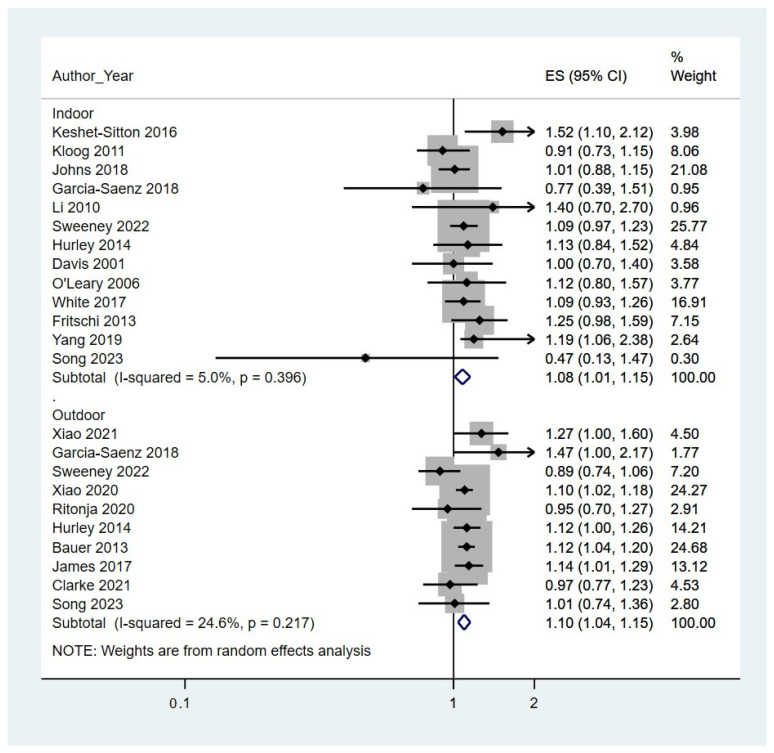
The effect size (ES) and 95% confidence interval (CI) of the association between light at night exposure and breast cancer risk for both indoor and outdoor exposure of *n* = 19 [25,26,27,28,29,32,33,34,35,41,43,44,47,48,50] studies, with *n* = 4 [30,31,42,45] assessing both. The solid diamond represents the estimated risk ratio of the highest exposure group, and the solid line represents the 95% CI. The dark grey square represents the study weight. The empty diamond represents the pooled risk ratio for each exposure group.

In our meta-analysis of the relationship between breast cancer risk and exposure to LAN, when comparing the highest versus the lowest LAN exposure categories, we found an overall positive association with breast cancer risk (summary RR, 1.09; 95% CI, 1.05–1.14). This pattern is further reflected in studies that specifically assessed outdoor LAN exposure (summary RR, 1.10; 95% CI 1.04–1.15) as well as subjective indoor LAN exposure (summary RR, 1.08; 95% CI, 1.01–1.15) (Figure 2). There was low study heterogeneity for both indoor (I^2^ = 5%) and outdoor (I^2^ = 25%) LAN exposures [24]. The associations of LAN with breast cancer risk were no different for case–control (indoor LAN, summary RR, 1.11; 95% CI 0.96–1.29; outdoor LAN, summary RR, 1.09; 95% CI, 0.86–1.39) or prospective cohort (indoor LAN, summary RR, 1.07; 95% CI 0.99–1.15; outdoor LAN, summary RR, 1.09; 95% CI, 1.01–1.17) studies. Likewise, the associations were unchanged when we excluded the one study with critical risk of bias (indoor LAN, summary RR, 1.08; 95% CI 1.02–1.15; outdoor LAN, summary RR, 1.10; 95% CI, 1.04–1.16) [42].

### 3.2. Other Cancers

We were unable to perform meta-analyses for other cancers because few studies have examined the association between LAN exposure and other cancer types. Table 2 describes the eight non-breast cancer studies that fit our search criteria. We identified two studies that examined the association between outdoor LAN exposure and risk of colorectal cancer, although they measured LAN using different modalities [46,49]. Using a job exposure matrix, Walasa et al. estimated that ≥7.5 years of shiftwork with LAN exposure was not associated with colorectal cancer risk compared with working no shifts with LAN exposure (OR, 0.91; 95% CI, 0.55–1.53) [49]. Garcia-Saenz et al. [46], using satellite light data, also reported no significant association between visual LAN exposure and colorectal cancer risk (OR, 0.9; 95% CI, 0.7–1.1); however, exposure to outdoor blue light exposure was associated with increased risk of colorectal cancer (OR, 1.7; 95% CI, 1.3–2.3). One study investigated prostate cancer risk with regard to LAN exposure and found mixed results. Specifically, while indoor LAN exposure assessed using a subjective measure of darkness showed a strong positive association with prostate cancer risk (OR, 2.79; 95% CI, 1.55–5.05), the association between outdoor LAN and risk of prostate cancer varied depending on the type of light (visual light OR, 0.56; 95% CI, 0.38–0.84; blue light OR, 2.05; 95% CI, 1.38–3.03) [45]. In three separate studies, individuals in the highest quintile of outdoor LAN had increased risks of non-Hodgkin’s lymphoma (HR, 1.32; 95% CI, 1.05–1.66), pancreatic cancer (HR, 1.24; 95% CI, 1.03–1.49), and thyroid cancer (HR, 1.55; 95% CI, 1.18–2.02) compared with individuals in the lowest quintile of outdoor LAN [38,39,40]. Finally, two separate studies reported no association between outdoor LAN exposure and the risks of liver cancer and endometrial cancer [36,37]. 

## 4. Discussion

We conducted a systematic review and, for breast cancer risk, a meta-analysis of the published literature on the association between LAN exposure and the risk of cancer development. We identified 26 studies (13 case–control and 13 cohort) that directly addressed this issue. In the meta-analysis of 19 breast cancer studies, there was a modest increased risk associated with high LAN exposure. The association was similar whether LAN exposure was from indoor or outdoor sources. Except for breast cancer, there was a paucity of site-specific cancer studies.

There is increasing focus on the potential for environmental exposures to influence a person’s risk of developing cancer. Prior meta-analyses, for example, of air pollution [51,52] and smoking [53,54], have demonstrated a link with increased risk of cancer in these exposure groups. LAN exposure has been under increasing scrutiny as a potentially harmful environmental health exposure, with several meta-analyses noting its association with obesity [55], diabetes [56], and breast cancer [17,18,19]. In our systematic review, we focused on the important intersection between the environmental health exposure of LAN and cancer risk. In addition to looking at multiple cancer sites, our findings build on the most recent prior meta-analysis of breast cancer risk by incorporating two new studies. Since the most recent meta-analysis of LAN exposure and breast cancer risk was published in late 2021 by Urbano et al. [17], two new studies have been published regarding breast cancer risk and LAN exposure that fit the search criteria of our analysis [31,42]. The addition of these two studies further supports the conclusions of the previous meta-analyses that show a moderate positive association between LAN exposure and breast cancer risk in women. When stratified by indoor versus outdoor LAN exposure, our analysis further supports the findings of Urbano. That is, regardless of sources, LAN is associated with breast cancer risk. The low statistical heterogeneity between studies in our analysis emphasizes this positive relationship by indicating a low likelihood that the consistency of evidence found is due to chance. Nonetheless, there remains a relative paucity of studies assessing the important relationship between LAN exposure and breast cancer risk. The continued positive association between the two calls for further analysis of different study populations of interest as well as an investigation into the mechanism of this association.

The dearth of studies examining the association between LAN exposure and cancer risk extends beyond breast cancer to other cancer types. Only eight studies meeting our search criteria examined associations of LAN with non-breast cancers, with only colorectal cancer having been examined in more than one study. The wide range of results, such as studies of prostate and thyroid cancers showing a strong positive relationship with LAN exposure [38,46] and liver and endometrial cancers showing no appreciable association [36,37], demonstrates an interesting potential for LAN to also be positively associated with other (non-breast) cancer types. The extreme shortage of studies, however, prevents any definitive conclusions from being made. While some studies speculate the impairment of melatonin and estrogen release due to LAN exposure playing an important role in breast cancer [57], impaired sleep quality can also impact oxidative stress and inflammatory levels, cause metabolic and circadian disruptions, or affect other hormonal secretions [14,57,58,59]. Inflammatory states and metabolic and circadian disruptions are known carcinogens [60,61], so it follows that LAN may affect cancer types beyond breast cancer. Animal studies also further support the relationship between LAN exposure and other cancer types [61,62]. Therefore, more investigation is needed to assess if there is a similar increase in cancers such as liver, colorectal, or prostate cancer when exposed to increased LAN levels. 

It is important to note the inherent differences in mechanisms of exposure to indoor and outdoor light. Of the 16 studies using outdoor light as a method of LAN exposure, all used satellite data as the source of outdoor LAN exposure; 13 of 16 studies utilized the U.S. Defense Meteorological Satellite Program’s Global Radiance Calibrated Night-time Lights high-dynamic range data, which had the benefit of assigning radiance units (nW/cm^2^/sr) to geocoded addresses. Two of the 16 studies, both in Spain, utilized ISS images to predict luminance and blue light exposure for each pixel, which was then geocoded. The final study assessing outdoor LAN utilized the New World Atlas of Artificial Night Sky Brightness to measure luminance on a grid to assign to resident addresses [11]. The use of satellite-gathered information has many advantages, including the ability to standardize luminance levels across large swaths of land, geocode the luminance to specific residence addresses, and easily analyze data. However, satellite data cannot fully reflect the true amount of LAN exposure an individual at a certain address may receive. Tree cover, building height, the ability of outdoor light to actually penetrate an individual’s sleeping environment, and local lighting levels undetectable by satellite imagery can greatly affect LAN exposure. Urban saturation of LAN also may distort the ability of satellite imagery to accurately measure LAN based on the limited resolution of images.

Indoor measurements of LAN are also an imperfect assessment of complete LAN exposure. Indoor LAN can reflect the true exposure of an individual to LAN exposure to a greater degree than outdoor LAN, as the vast majority of individuals are indoors at night. However, accurately measuring indoor LAN can be difficult. The 14 studies in our analysis of both breast and non-breast cancers all administered a questionnaire to assess indoor LAN exposure. Some of the questionnaires used an iteration of a three- or four-point scale to quantify the level of indoor LAN by asking the participant questions, such as if they could see their hand in front of their face, if they could see across the room, or if they could read a book comfortably. Other yes and no questions were often included in the questionnaire, such as if the participant used a nightlight or had a TV or other strong source of light turned on during the night. The reliability of using subjective recall of light at night exposure, especially when participants were asked to remember their exposure for the preceding decade, is questionable, and it is unclear if this was able to be standardized across studies. Validation studies comparing with the objective measurements above are needed before definitive conclusions can be drawn from observational studies of LAN exposure.

This review of the effects of LAN on cancer risk has several strengths and limitations. Our study validates and supports previous studies [17,18,19] exploring the overall relationship between LAN exposure and breast cancer risk while also being the first study to synthesize the evidence around the association of LAN exposure with the risk of other cancer types. We provide a solid framework to continue investigations into the effects of LAN on cancer types other than breast cancer. As discussed before, the inherent limitations regarding the measurement of LAN exposure present fluctuations in an unclear direction depending on whether the exposure was indoor or outdoor. Indoor measurements of LAN were more reflective of true LAN exposure but were subject to recall and measurement bias, while outdoor measures of LAN are more accurately measurable but are subject to wide variability in their ability to reach participants at night. Next, while the association with breast cancer risk was found with both case–control and prospective cohort studies, better evidence for causality comes from prospective cohort studies [63]. At the moment, the Bradford Hill criteria for causality [64] between LAN exposure and breast cancer that are well discussed in the literature are consistency, specificity, and the biological gradient. However, biological plausibility and coherence could be strengthened through biochemical research on sleep, hormonal, and cancer pathways. Temporality can be further investigated through future studies ascertaining LAN exposure prior to cancer development as well as exposure to LAN across the lifetime. Our meta-analysis was also limited by the fact that the combined effect of LAN was calculated by incorporating different maximum exposure definitions, some categorical and some with a numerical percentile cutoff. There are also potential confounders that were unaccounted for among some or all of the studies, such as additional environmental exposures like noise or occupational status. However, most studies controlled for the major risk factors for breast cancer like parity, obesity, age of menopause, and smoking status. For non-breast cancer studies, the scarcity of analyses prevents any strong conclusions from being made regarding the impact of LAN on other cancer risks and requires additional effort in the future to elucidate the nuances of this relationship. This also limits the generalizability of our observations regarding non-breast cancer studies. More studies investigating the association between LAN exposure and cancer risk need to be performed for other cancer types, specifically for cancers such as endometrial, ovarian, and prostate that are also highly hormonally regulated, like breast cancer [65]. In line with this, biochemical research that further elucidates mechanisms of action between sleep, hormonal axis regulation, and oncogenesis can work in tandem with population-based studies such as ours to further paint a clearer picture of the role sleep has on cancer incidence.

## 5. Conclusions

Increasing urbanization and the ubiquity of LAN across more and more of the global population present a growing public health concern regarding cancer incidence. Our study found a positive association between both indoor and outdoor LAN exposures and breast cancer risk. We also highlight the lack of information regarding the association between LAN exposure and the risks of non-breast cancers. We suggest more investigation be conducted regarding the specific mechanisms between LAN exposure and cancer risk as well as analysis on specific groups that may be at risk for other cancer types. Further elucidation of these relationships may inform public health and governmental policy regarding LAN to reduce the global burden of cancer.

## Figures and Tables

**Table 1:** Descriptions of studies and their study designs.

First Author and Year	Cancer Type	LAN Assessment	Study Design
Davis, 2001 [35]	Breast	Indoor	Case–Control
O’Leary, 2006 [34]	Breast	Indoor	Case–Control
Li, 2010 [33]	Breast	Indoor	Case–Control
Kloog, 2011 [44]	Breast	Indoor	Case–Control
Fritschi, 2013 [50]	Breast	Indoor	Case–Control
Keshet-Sitton, 2016 [43]	Breast	Indoor	Case–Control
White, 2017 [32]	Breast	Indoor	Prospective Cohort
Johns, 2018 [47]	Breast	Indoor	Prospective Cohort
Yang, 2019 [41]	Breast	Indoor	Case–Control
Hurley, 2014 [30]	Breast	Indoor and Outdoor	Prospective Cohort
Garcia-Saenz, 2018 [45]	Breast	Indoor and Outdoor	Case–Control
Sweeney, 2022 [31]	Breast	Indoor and Outdoor	Prospective Cohort
Song, 2023 [42]	Breast	Indoor and Outdoor	Case–Control
Bauer, 2013 [29]	Breast	Outdoor	Case–Control
James, 2017 [25]	Breast	Outdoor	Prospective Cohort
Ritonja, 2020 [28]	Breast	Outdoor	Case–Control
Xiao, 2020 [27]	Breast	Outdoor	Prospective Cohort
Clarke, 2021 [48]	Breast	Outdoor	Prospective Cohort
Xiao, 2021 [39]	Breast	Outdoor	Prospective Cohort
*n* = 19 studies			
Walasa, 2018 [49]	Colorectal	Indoor	Case–Control
Garcia-Saenz, 2018 [45]	Prostate	Indoor & Outdoor	Case–Control
Garcia-Saenz, 2020 [46]	Colorectal	Outdoor	Case–Control
Zhong, 2020 [40]	NHL	Outdoor	Prospective Cohort
Xiao, 2021 [26]	Pancreatic	Outdoor	Prospective Cohort
Zhang, 2021 [38]	Thyroid	Outdoor	Prospective Cohort
Park, 2022 [37]	Liver	Outdoor	Prospective Cohort
Medgyesi, 2023 [36]	Endometrial	Outdoor	Prospective Cohort
*n* = 8 studies			

**Table 2:** Full descriptions of non-breast cancer studies identified during the literature search.

Author and Year	Cancer Type	LAN Exposure	Study Design	Sampling Frame	Participant Source	LAN Assessment	Reference Group	Exposure	Adjusted OR/RR	Adjusted/Matched Factors
Walasa, 2018 [49]	Colorectal	Indoor	Case-Control	Population-based	Western Australia Bowel Health Study	Self-reported	No shifts with Light at night	7.5+ years of shiftwork with light at night	0.91 (0.55–1.53)	age, education, SES, smoking, and alcohol use
Garcia-Saenz, 2018 [45]	Prostate	Indoor	Case-Control	Population-based	MCC-Spain	Self-reported	Total Darkness	Very Illuminated	2.79 (1.55–5.04)	age, center, educational level, socioeconomic status, UVI, BMI, tobacco, family history of breast/prostate cancer, chronotype, menopausal status (breast cancer), and mutual adjustment for other light exposures
Garcia-Saenz, 2018 [45]	Prostate	Outdoor	Case-Control	Population-based	MCC-Spain	Measured	Outdoor LAN Q1	Outdoor LAN Q3	0.56 (0.38–0.84)	age, center, educational level, socioeconomic status, UVI, BMI, tobacco, family history of breast/prostate cancer, chronotype, menopausal status (breast cancer), and mutual adjustment for other light exposures
						Measured	Outdoor Blue LAN Q1	Outdoor Blue LAN Q3	2.05 (1.38–3.03)	
Garcia-Saenz, 2020 [46]	Colorectal	Outdoor	Case-Control	Population-based	MCC-Spain	Measured	Outdoor LAN Q1	Outdoor LAN Q3	0.9 (0.7–1.1)	Area, age, sex, educational level, WCRF score, Urban Vulnerability Index, family history, smoking habits, sleeping problems, and sleep duration
						Measured	Outdoor Blue LAN Q1	Outdoor Blue LAN Q3	1.7 (1.3–2.3)	
Zhong, 2020 [40]	NHL	Outdoor	Prospective Cohort	Population-based	California Teachers Study	Measured	Outdoor LAN Q1	Outdoor LAN Q5	1.32 (1.05–1.66)	age, race, SES, BMI, smoking, alcohol, and FH of NHL
Xiao, 2021 [39]	Pancreatic	Outdoor	Prospective Cohort	Population-based	NIH-AARP	Measured	Outdoor LAN Q1	Outdoor LAN Q5	1.24 (1.03–1.49)	Age, sex, race, education, marital status, state of residence, median home value, poverty rate, and population density at the census tract level
Zhang, 2021 [38]	Thyroid	Outdoor	Prospective Cohort	Population-based	NIH-AARP	Measured	Outdoor LAN Q1	Outdoor LAN Q5	1.55 (1.18–2.02)	Age, sex, race, education, marital status, state of residence, median home value, poverty rate, and population density at the census tract level
Park, 2022 [37]	Liver	Outdoor	Prospective Cohort	Population-based	NIH-AARP	Measured	Outdoor LAN Q1	Outdoor LAN Q5	0.96 (0.77–1.20)	age, sex, race/ethnicity, education, BMI, diabetes, aspirin use, coffee consumption, night-time sleep duration, state, income, and urban–rural code
Medgyesi, 2023 [36]	Endometrial	Outdoor	Prospective Cohort	Population-based	NIH-AARP	Measured	Outdoor LAN Q1	Outdoor LAN Q5	0.93 (0.77–1.1)	Age, race/ethnicity, poverty, state, BMI, years of OC use, age at menopause, HRT use, parity, PM2.5, and metro-rural area

## Data Availability

This study used data obtained from public online repositories.

## Supplementary Materials

The following supporting information can be downloaded at https://www.mdpi.com/article/10.3390/cancers16152653/s1, Table S1: Details of search strategy with the search strings used in PubMed, Web of Science, and Embase databases; Table S2: Description of the domains used by the Risk of Bias in Non-Randomized Studies tool; Table S3: Full details of the studies included our analysis; Table S4: Consensus results of the Risk of Bias in Non-randomized Studies analysis.
