# Supplementary material for: Exposure to Light at Night and Risk of Cancer: A Systematic Review, Meta-Analysis, and Data Synthesis
Source: Cancers (Basel). 2024 Jul 26;16(15):2653. doi: 10.3390/cancers16152653 (PMC11311462; doi:10.3390/cancers16152653)
Supplement: Supplementary file 1 [file cancers-16-02653-s001.zip › cancers-3042213-supplementary.pdf]

Supplementary Table S1. Details of search strategy with the search strings used in PubMed, Web of Science, and Embase databases.

Database search	Search string	Results (n)
**PubMed**	("Neoplasms"[Mesh] OR "cancer" [tiab] OR "neoplasm"[tiab]) AND ("Light/adverse effects"[Mesh] OR "Lighting/adverse effects"[Mesh] OR "Light at night"[tiab] OR “Light exposure at night”[Mesh])	7,422
**Web of Science**	(TS=("light at night") OR TI=("light at night") OR AB=("light at night")) AND (TS=("cancer") OR TI=("cancer") OR AB=("cancer"))	365
**Embase**	('cancer'/exp OR 'cancer':ti,ab,kw OR 'neoplasm':ti,ab,kw) AND ('light at':ti,ab OR 'lightning'/exp OR 'lightning':ti,ab,kw OR 'artificial light'/exp)	1,131

Supplementary Table S2. Description of the domains used by the Risk of Bias in Non-Randomized Studies tool.

Domains	Criteria
Bias due to confounding	Factor considered mandatory to judge a study at moderate risk of bias was age and not controlling for other disruptors of circadian rhythm. Factors considered mandatory to judge a study at low risk of bias are at least two among: hormone replacement therapy use, family history of breast cancer, prior radiation exposure, or smoking.
Bias in selection of participants into the study	Selection of eligible participants must not be related to lightning exposure to be at low risk of bias. Moderate risk was considered for studies with a small sample size or non-generalizable demographic makeup. Non-random selection of participants was considered high risk.
Bias in classification of exposures	Possible exposure misclassification with moderate risk of bias for studies that relied on self-report for exposure assessment only, high risk of bias if the questionnaire was not validated or self-administrated (i.e., without a trained interviewer).
Bias due to deviations from intended exposures	Studies are considered at low risk of bias if participants are analyzed based on the exposure group they are assigned at the beginning of the study, while are considered at high risk if changing in exposure classification occurred during the study.
Bias due to missing data	10% was considered as reasonable cut off point for missing data to be considered a study at low risk of bias in both case-control and cohort studies.
Bias in measurement of the outcome	Possible bias based on the modality of outcome assessment. High risk in case of assessment based on self-report only, without external validation, moderate with validation.
Bias in selection of the reported result	For low risk of bias clear evidence that results have not been selected must be present as well as clear and complete reporting of statistical methods.
Overall risk bias	Overall risk bias was determined subjectively by reviewers and agreed upon by consensus based on the severity of the cumulative effects of each domain on the overall risk of bias of the study

Supplementary Table S3. Full details of the studies included our analysis

Author & Year	Cancer Type	LAN Exposure	Study Design	Sampling Frame	Participant Source	LAN assessment	Reference group	Exposure	Adjusted OR/RR	Adjusted/Matched Factors
Walasa 2018	Colorectal	Indoor	Case-Control	Population-based	Western Australia Bowel Health Study	Self-reported	No shifts with Light at night	7.5+ years of shiftwork with light at night	0.91 (0.55 - 1.53)	age, education, SES, smoking, alcohol use,
Garcia-Saenz 2018	Prostate	Indoor	Case-Control	Population-based	MCC-Spain	Self-reported	Total Darkness	Very Illuminated	2.79 (1.55-5.04)	age, center, educational level, socioeconomic status, UVI, BMI, tobacco, family history of breast/prostate cancer, chronotype, menopausal status (breast cancer), and mutual adjustment for other light exposures
Garcia-Saenz 2018	Prostate	Outdoor	Case-Control	Population-based	MCC-Spain	Measured	Outdoor LAN Q1	Outdoor LAN Q3	0.56 (0.38-0.84)	age, center, educational level, socioeconomic status, UVI, BMI, tobacco, family history of breast/prostate cancer, chronotype, menopausal status (breast cancer), and mutual adjustment for other light exposures
						Measured	Outdoor Blue LAN Q1	Outdoor Blue LAN Q3	2.05 (1.38-3.03)	

Author & Year	Cancer Type	LAN Exposure	Study Design	Sampling Frame	Participant Source	LAN assessment	Reference group	Exposure	Adjusted OR/RR	Adjusted/Matched Factors
Garcia-Saenz 2020	Colorectal	Outdoor	Case-Control	Population-based	MCC-Spain	Measured	Outdoor LAN Q1	Outdoor LAN Q3	0.9 (0.7-1.1)	Area, age, sex, educational level, WCRF score, Urban Vulnerability Index, family history, smoking habits, sleeping problems, sleep duration
Garcia-Saenz 2018	Prostate	Outdoor	Case-Control	Population-based	MCC-Spain	Measured	Outdoor LAN Q1	Outdoor LAN Q3	0.56 (0.38-0.84)	age, center, educational level, socioeconomic status, UVI, BMI, tobacco, family history of breast/prostate cancer, chronotype, menopausal status (breast cancer), and mutual adjustment for other light exposures
						Measured	Outdoor Blue LAN Q1	Outdoor Blue LAN Q3	2.05 (1.38-3.03)	
Garcia-Saenz 2020	Colorectal	Outdoor	Case-Control	Population-based	MCC-Spain	Measured	Outdoor LAN Q1	Outdoor LAN Q3	0.9 (0.7-1.1)	Area, age, sex, educational level, WCRF score, Urban Vulnerability Index, family history, smoking habits, sleeping problems, sleep duration
						Measured	Outdoor Blue LAN Q1	Outdoor Blue LAN Q3	1.7 (1.3-2.3)	

Author & Year	Cancer Type	LAN Exposure	Study Design	Sampling Frame	Participant Source	LAN assessment	Reference group	Exposure	Adjusted OR/RR	Adjusted/Matched Factors
Zhong 2020	NHL	Outdoor	Prospective Cohort	Population-based	California Teachers Study	Measured	Outdoor LAN Q1	Outdoor LAN Q5	1.32 (1.05-1.66)	age, race, SES, BMI, smoking, alcohol, FH of NHL
Xiao 2021	Pancreatic	Outdoor	Prospective Cohort	Population-based	NIH-AARP	Measured	Outdoor LAN Q1	Outdoor LAN Q5	1.24 (1.03-1.49)	Age, sex race, education, marital status, state of residence, and median home value, poverty rate, and population density at the census-tract level.
Zhang 2021	Thyroid	Outdoor	Prospective Cohort	Population-based	NIH-AARP	Measured	Outdoor LAN Q1	Outdoor LAN Q5	1.55 (1.18-2.02)	Age, sex race, education, marital status, state of residence, and median home value, poverty rate, and population density at the census-tract level.
Park 2022	Liver	Outdoor	Prospective Cohort	Population-based	NIH-AARP	Measured	Outdoor LAN Q1	Outdoor LAN Q5	0.96 (0.77-1.20)	age, sex, race/ethnicity, education, BMI, diabetes, aspirin use, coffee consumption, nighttime sleep duration, state, income, urban-rural code

Author & Year	Cancer Type	LAN Exposure	Study Design	Sampling Frame	Participant Source	LAN assessment	Reference group	Exposure	Adjusted OR/RR	Adjusted/Matched Factors
Fritschi 2013	Breast	Indoor	Case-Control	Population-based	Breast Cancer Employment and Environment Study	Self-reported	Low indoor LAN	Easily read at night	1.25 (0.98-1.59)	age group adjusted
Keshet-Sitton 2016	Breast	Indoor	Case-Control	Clinical/Hospital	Israel-Jewish Population	Self-reported	Completely dark	Resides near strong artificial LAN sources	1.52 (1.10-2.12)	matched age and place, stepwise adjustment
White 2017	Breast	Indoor	Prospective Cohort	Population-based	Sister Study	Self-reported	No light while sleeping	Light/TV in a room	1.09 (0.93-1.26)	race, education, income, marital status, postmenopausal hormone use, use or oral contraceptives, alcohol consumption, age at menarche, parity, age at first birth, age at menopause, pack years of smoking, physical activity

Author & Year	Cancer Type	LAN Exposure	Study Design	Sampling Frame	Participant Source	LAN assessment	Reference group	Exposure	Adjusted OR/RR	Adjusted/Matched Factors
Johns 2018	Breast	Indoor	Prospective Cohort	Population-based	UK Generations Study	Self-reported	Complete indoor darkness	Can see across room	1.01 (0.88-1.15)	Cox proportional hazards regression with attained age as time scale, adjusted for: year of birth, history of benign breast disease, breast cancer in a first-degree relative, socioeconomic score, age at menarche, age at first birth, parity, duration of breastfeeding, oral contraceptive use, hormone replacement therapy use, menopausal status and age at menopause where applicable, pre-menopausal and post-menopausal body mass index, alcohol consumption, smoking and physical activity level
Yang 2019	Breast	Indoor	Case-Control	Population-based	Jiujiang Breast Cancer Study	Self-reported	Indoor Darkness	Can read at night	1.19 (1.06 - 2.68)	age, education, income, occupation, # of births, age of menarche, menopausal hormone use, marital status, smoking, alcohol, family history of BC, produce consumption, exercise, BMI, sleep quality

Author & Year	Cancer Type	LAN Exposure	Study Design	Sampling Frame	Participant Source	LAN assessment	Reference group	Exposure	Adjusted OR/RR	Adjusted/Matched Factors
Hurley 2014	Breast	Indoor	Prospective Cohort	Population-based	California Teachers Study	Measured	No indoor LAN	Heavy indoor LAN use	1.03 (0.90-1.18)	age, race, FH Bca, age at menarche, pregnancy history, breast feeding history, PA< BMI, alcohol, menopausal status, HRT, smoking, SES, urbanization
		Outdoor	Prospective Cohort	Population-based	California Teachers Study	Measured	Outdoor LAN Q1	Outdoor LAN Q5	1.12 (1.00-1.26)	age, race, FH Bca, age at menarche, pregnancy history, breast feeding history, PA< BMI, alcohol, menopausal status, HRT, smoking, SES, urbanization

Author & Year	Cancer Type	LAN Exposure	Study Design	Sampling Frame	Participant Source	LAN assessment	Reference group	Exposure	Adjusted OR/RR	Adjusted/Matched Factors
Garcia-Saenz 2018	Breast	Indoor	Case-Control	Population-based	MCC-Spain	Self-reported	Total Darkness	Very Illuminated	0.77 (0.39-1.51)	age, center, educational level, socioeconomic status, UVI, BMI, tobacco, family history of breast/prostate cancer, chronotype, menopausal status (breast cancer), and mutual adjustment for other light exposures
		Outdoor	Case-Control	Population-based	MCC-Spain	Measured	Outdoor LAN Q1	Outdoor LAN Q3	0.81 (0.54-1.20)	age, center, educational level, socioeconomic status, UVI, BMI, tobacco, family history of breast/prostate cancer, chronotype, menopausal status (breast cancer), and mutual adjustment for other light exposures
							Outdoor Blue LAN Q1	Outdoor Blue LAN Q3	1.47 (1.00-2.17)	

Author & Year	Cancer Type	LAN Exposure	Study Design	Sampling Frame	Participant Source	LAN assessment	Reference group	Exposure	Adjusted OR/RR	Adjusted/Matched Factors
Sweeney 2022	Breast	Indoor	Prospective Cohort	Population-based	Sister Study	Measured	No light on while sleeping	Light/TV in a room	1.09 (0.97-1.23)	Age, race/ethnicity, education, income, ADI, latitude, population density, PM2.5, NO2, green space, noise
		Outdoor	Prospective Cohort	Population-based	Sister Study	Measured	Outdoor LAN Q1	Outdoor LAN Q5	0.89 (0.74-1.06)	
Song 2023	Breast	Indoor	Case-Control	Population-based	Cancer Hospital of the Chinese Academy of Medical Sciences	Self-reported	No bedroom brightness	High bedroom brightness	0.47 (0.13 - 1.47)	age, BMI, smoking, alcohol, menopause status, family history of BC, duration of breastfeeding, age of menarche, # of pregnancies, age of first pregnancy, oral contraceptive use, hormone therapy use
		Outdoor	Case-Control	Population-based	Cancer Hospital of the Chinese Academy of Medical Sciences	Self-reported	No outdoor light while sleeping	Outdoor light while sleeping	1.01 (0.74 - 1.36)	
Bauer 2013	Breast	Outdoor	Other	Population-based	Georgia	Measured	Outdoor LAN Q1	Outdoor LAN Q3	1.12 (1.04-1.20)	race, tumor grade and stage, year of diagnosis, age at cancer diagnosis, Metropolitan Statistical Area (MSA) status, births per 1,000 women aged 15–50, MSA population mobility, population over 16 in the labor force, and prevalence of cigarette smoking

Author & Year	Cancer Type	LAN Exposure	Study Design	Sampling Frame	Participant Source	LAN assessment	Reference group	Exposure	Adjusted OR/RR	Adjusted/Matched Factors
James 2017	Breast	Outdoor	Prospective Cohort	Population-based	Nurses' Health Study II	Measured	Outdoor LAN Q1	Outdoor LAN Q5	1.14 (1.01-1.29)	benign breast disease history, family history of breast cancer, age at menarche, parity and age at first birth, height, white race, BMI, BMI at age 18, oral contraceptive use, mammography screening, menopausal status, smoking status, alternative healthy eating index, physical activity, marital status, living alone, personal income, shift work after 1989, region, PM2:5, census-tract median home value, income, and population density
Ritonja 2020	Breast	Outdoor	Case-Control	Population-based	Vancouver, British Columbia, and Kingston, Ontario	Measured	Outdoor LAN Q1	Outdoor LAN Q3	0.95 (0.70-1.27)	Age, ethnicity, menopausal status, FH Bca, age at menarche, BMI, income, education, parity and age at first birth, years OC us, screening, smoking, night work, alcohol, population density, neighborhood income
Xiao 2020	Breast	Outdoor	Prospective Cohort	Population-based	NIH-AARP	Measured	Outdoor LAN Q1	Outdoor LAN Q5	1.10 (1.02-1.18)	Fully adjusted

Author & Year	Cancer Type	LAN Exposure	Study Design	Sampling Frame	Participant Source	LAN assessment	Reference group	Exposure	Adjusted OR/RR	Adjusted/Matched Factors
Clarke 2021	Breast	Outdoor	Prospective Cohort	Population-based	Danish Nurse Cohort	Measured	Outdoor LAN Q1	Outdoor LAN Q3	0.97 (0.77-1.23)	age, year, birth cohort, urbanicity, alcohol, marital status, night shirt work, latitude
Xiao 2021	Breast	Outdoor	Prospective Cohort	Clinical/Hospital	Southern Community Cohort Study	Measured	Outdoor LAN Q1	Outdoor LAN Q5	1.27 (1.00-1.60)	Age, education, marital status, income, health insurance coverage, smoking, family history of breast or ovarian cancer among first-degree female relatives, mammogram, age at menarche, postmenopausal status, ever use of menopausal hormone therapy, average number of alcoholic drinks consumed per day, and population density and percentage of households living under the 2000 federal poverty line in the census tract

Supplementary Table S4. Consensus results of the Risk of Bias in Non-randomized Studies analysis.

**Paper Evaluated**	**Confounding**	**Selection**	**Measurement of exposure**	**Departures from exposure**	**Missing Data**	**Measurement of Outcomes**	**Reported Results**	**Study - level RoB judgement**
Davis 2001	low	low	moderate	low	low	low	low	low
O'Leary 2006	low	moderate	moderate	low	low	low	low	low
Li 2010	low	moderate	low	low	low	low	low	low
Kloog 2011	moderate	moderate	low	low	low	low	low	low
Fritschi 2013	low	low	moderate	low	low	low	low	low
Keshet-Sitton 2016	moderate	serious	moderate	low	low	low	low	moderate
White 2017	low	low	low	low	low	low	low	low
Johns 2018	moderate	low	low	low	low	low	low	low
Yang 2019	low	moderate	low	low	low	low	low	low
Hurley 2014	low	moderate	low	low	low	low	low	low
Garcia-Saenz 2018	low	low	low	low	low	low	low	low
Sweeney 2022	low	low	low	low	low	low	low	low
Song 2023	moderate	serious	serious	low	low	low	low	serious
Bauer 2013	low	low	moderate	low	low	low	low	low
James 2017	low	moderate	moderate	low	low	low	low	low
Ritonja 2020	low	low	low	low	low	low	low	low
Xiao 2020	moderate	low	moderate	low	low	low	low	low
Clarke 2021	moderate	moderate	moderate	low	low	low	low	moderate
Xiao 2021 (Breast Cancer)	low	low	moderate	low	low	low	low	low
Walasa 2018	moderate	serious	low	low	low	low	low	moderate
Garcia-Saenz 2020	moderate	low	moderate	low	low	low	low	low
Zhong 2020	low	moderate	moderate	low	low	low	low	low
Zhang 2021	low	low	low	low	low	low	low	low
Park 2022	low	low	low	low	low	low	low	low
Medgyesi 2023	low	low	low	low	low	low	low	low
Portnov 2016	low	low	low	low	low	low	low	low
Xia 2021 (Pancreatic Cancer)	low	low	low	low	low	low	low	low
**Item Level Judgment**	**low**	**moderate**	**moderate**	**low**	**low**	**low**	**low**	**low**
